# Sustained Release of Risedronate from PLGA Microparticles Embedded in Alginate Hydrogel for Treatment of Bony Lesions

**DOI:** 10.52547/ibj.26.2.124

**Published:** 2021-11-14

**Authors:** Ghazaleh Azari, Shabnam Aghayan, Ehsan Seyedjafari

**Affiliations:** 1 Faculty of Dentistry, Tehran Medical Sciences, Islamic Azad University, Tehran, Iran;; 2 Department of Periodontology, Faculty of Dentistry, Tehran Medical Sciences, Islamic Azad University, Tehran, Iran;; 3 Department of Biotechnology, College of Sciences, University of Tehran, Tehran, Iran

**Keywords:** Alginates, Hydrogels, Polylactic acid-polyglycolic acid copolymer, Risedronic acid

## Abstract

**Background:**

Inflammatory bone resorption in periodontitis can lead to tooth loss. Systemic administration of bisphosphonates such as risedronate for preventing bone resorption can cause adverse effects. ALG and PLGA microparticles have been studied as drug delivery systems for sustained release of drugs. Therefore, the release pattern of risedronate from PLGA microparticles embedded with ALG was studied as a drug delivery system for sustained release of the drug, which can be used in local administrations.

**Methods:**

Risedronate-containing PLGA microparticles were fabricated using double emulsion solvent evaporation technique. Ionic cross-linking method was used to fabricate risedronate-loaded ALG. Risedronate-containing PLGA microparticles were then coated with ALG. The calibration curve of risedronate was traced to measure EE and study the release pattern. SEM imaging was carried out, and cell toxicity was examined using MTT assay. Statistical analysis of data was carried out using SPSS ver. 20 software, via one-way ANOVA and Tukey’s tests.

**Results:**

SEM imaging showed open porosities on ALGs. The mean EE of PLGA microparticles for risedronate was 57.14 ± 3.70%. Risedronate released completely after 72 h from ALG, and the cumulative release was significantly higher (*p* = 0.000) compared to PLGA microspheres coated with ALG, which demonstrated sustained released of risedronate until day 28. Risedronate-loaded ALG showed a significant decrease in gingival fibroblasts cell viability (*p* < 0.05).

**Conclusion:**

Alginate-coated PLGA microspheres could release risedronate in a sustained and controlled way and also did not show cell toxicity. Therefore, they seem to be an appropriate system for risedronate delivery in local applications.

## INTRODUCTION

Antiresorptive agents such as bisphosphonates are generally used to decrease bone turnover. In addition, they are applied for the treatment of several bone disorders, including osteoporosis, tumor-associated osteolysis, arthritis, and periodontitis^[^^1^^]^.

In 1998, risedronate was first approved to treat Paget’s disease in USA^[^^2^^]^. Risedronate is a nitrogen-containing bisphosphonate that exerts its antiresorptive effects by attaching to hydroxy apatite in bone tissue, as well as by inhibiting the activity of osteoclasts and inducing apoptosis in these cells^[^^3^^]^.

Bisphosphonate-induced osteonecrosis of the jaw, which develops in systemic applications, is a significant side effect that affects the patients receiving these drugs^[^^4^^]^. Oral bisphosphonates can cause recurring ulcers and a burning sensation in mouth, inflammation of the esophagus, peptic ulcers, and abdominal pain^[^^5^^]^. Orally administrated risedronate can lead to acidic reflux, and acute phase reactions, as well as muscle and bone pain^[^^6^^]^. Occurrence of such adverse effects during systemic administration of these drugs has shown a great necessity for their local application^[^^7^^]^. 

Biocompatible synthetic polyesters such as polylactic acid and polyglycolic acid have been used as absorbable sutures and monofilaments since early 1970s. Among the copolymers of these materials, PLGA has several therapeutic applications because of its biocompatibility, biodegradability, and the ability of sustained drug release^[^^8^^]^. Single and double (multiple) emulsion methods are two of PLGA fabrication approaches^[^^9^^]^. 

Hydrogels are special kinds of gels that consist of hydrophilic polymer chains and are capable of retention and absorption of large amounts of water; for this reason, their application as drug delivery carriers has received significant attention in medicine^[^^10^^]^. Hydrogels were first successfully used in the fabrication of contact lenses by Lim and Wichtrele^[^^11^^]^ in 1960s.

Alginate is a natural polysaccharide extracted from brown algae and has applications in food and drug industries. Due to the biocompatibility, biodegradability and non-immunogenicity features, alginate has various biomedicine applications, including tissue engineering and drug delivery. It is also used as an additive to drug formulations to prevent gastric reflux. In 1881, algic acid was characterized and extracted with sodium carbonate; the alginate was later precipitated out of solution at low pH^[^^12^^]^.

Regarding the drug delivery, alginate is used for the controlled and local delivery of the drug. As hydrated alginate creates a gelatin layer, which acts as a barrier against rapid release of the drug^[^^13^^,^^14^^]^, its application for controlled, slow, and sustained drug delivery is noteworthy in drug delivery systems^[^^15^^,^^16^^]^.

There has been little study regarding the local application of risedronate with hydrogel-biopolymer carriers. The result of the present study can assist in finding a simple and cost-effective solution for the local application of risedronate; therefore, we can benefit from the drug in improving bony lesions, while avoiding the systemic side effects and also increasing the patient’s compliance.

## MATERIAL AND METHODS

**Materials**

Sodium alginate, PLGA (50:50, RG-504H), risedronate, chloroform, L-glutamine, and polyvinyl alcohol were purchased from Sigma (St. Louis, MO, USA). CaCl_2_ and DMSO were obtained from Merck (Germany). HGF1-P1 was acquired from Pasteur Institute of Iran (Tehran). DMEM and fetal bovine serum were procured from Gibco (USA).

**Fabrication of PLGA microspheres**

**Fig. 1:**
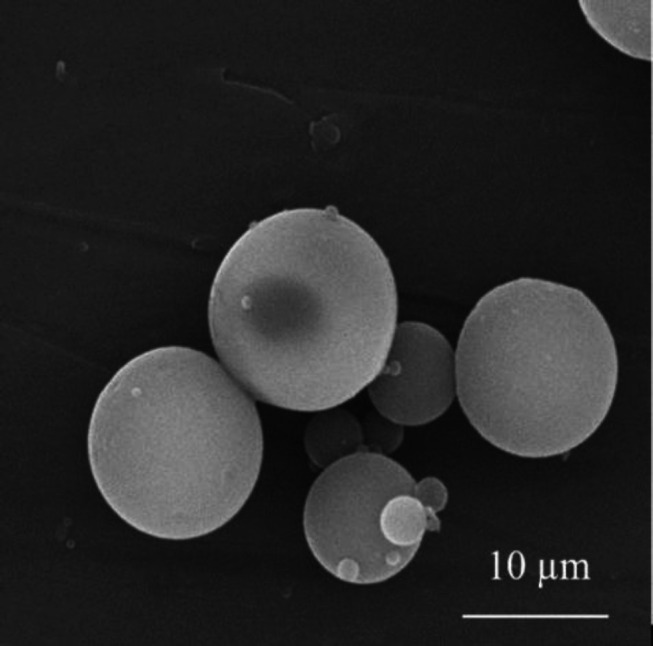
Field emission scanning electron micrograph of PLGA microparticles

PLGA microspheres were fabricated using double emulsion solvent evaporation method^[^^16^^]^. For the preparation of the organic phase of PLGA 10% solution, 0.1 g of PLGA was solved in a total volume of 1 mL of chloroform, using a magnetic stirrer (Heidolph, Germany) for 1 hour. Next, 1 mL of W_1_ phase (2% w/v polyvinyl alcohol) was prepared and added to 1 mL of PLGA solution. This compound (W_1_/O phase) was stirred using a homogenizer (IKA, Germany) at 13,000 ×g for 1 min. To prepare the secondary emulsion solution, we added the W_1_/O phase solution to 30 mL of 2% w/v polyvinyl alcohol solution (W_1_/O/W_2 _phase). To evaporate the solvent and hardening of the microspheres, the double emulsion solution was stirred at 25 °C for 2 hours and then centrifuged at 25,000 ×g for 10 minutes, in order to collect the microspheres. After the complete deposition of microspheres, the resultant microparticles were washed three times with distilled water using a centrifuge and then transferred to a freeze dryer (Martin Christ, Germany) at -15 °C temperature. Following complete solidification, the microparticles were lyophilized at -20 °C for 48 hours and at 0.5 bar pressure. The microspheres were then collected and kept at 0 °C temperature (Fig. 1).

**Fabrication of PLGA microspheres loaded with risedronate**

Fabrication of PLGA microspheres loaded with risedronate was performed by adding 2 mg of risedronate to W_1 _phase solution (2% w/v polyvinyl alcohol) during making PLGA microspheres using double emulsion solvent evaporation method as metoned ablove. 

**Fabrication of** **ALG** **, risedronate-loaded** **hydrogel** **, and** **ALG** **hydrogel with embedded PLGA microspheres**

For the preparation of 2% w/v alginate solution, 2 g of alginate was dissolved in 100 mL of double distilled water. Then 100 mM of CaCl_2_ was added to alginate solution to achieve the final solution. In order to prepare risedronate-loaded ALG hydrogel and ALG hydrogel with embedded risedronate-containing PLGA microspheres, we added 2 mg of risedronate and 14 mg of risedronate-containing PLGA solution to 1 mL of alginate solution, respectively. 

**SEM imaging**

The freeze-dried samples were cut at 1 cm × 1 cm dimension, and after being placed on an aluminum foil, they were coated with a thin layer of gold. Then the images of the desired samples were taken by a scanning electron microscope (Seron Technologies, South Korea).

**Preparation of risedronate calibration curve**

Risedronate was dissolved in PBS in 11 different concentrations and the absorbance of each concentration was measured at 262 nm using a UV-Vis spectrophotometer (Perkin Elmer, USA). The risedronate calibration curve was created subsequently^[^^17^^]^.

**Determination of EE in PLGA microspheres**

Samples were first added to 0.1 M of NaOH^[^^18^^]^ and then agitated by magnetic stirring for 16 hours, with the purpose of complete degradation of microspheres. The resultant solution was finally centrifuged at 13,000 ×g at 25 °C for 10 minutes. The supernatant was then collected, and its risedronate content was determined by UV spectroscopy as mentioned above. The EE was finally calculated using the following formula:

EE% = (W_t_/W_i_) ×100%

W_t_ is the total amount of loaded drug, and W_i_ is the total quantity of drug added initially during preparation. The steps discussed were repeated five times, and the results were reported as mean ± SD.

**Examining release behavior**

To examine the release of risedronate, 14 mg of the prepared samples were added to 3 ml of PBS. The containers were then placed in an incubator shaker (Alfa, Iran) at 100 rpm at 37 °C. The PBS was collected from each well and replaced with a fresh PBS at predetermined time intervals. The release of risedronate was determined by UV-Vis spectrophotometry at 262 nm. The absorbance was converted to concentration using risedronate calibration curve. The cumulative release was then calculated using the following equation^[^^19^^]^:

$\left(\frac{M_{t}}{M_{\infty }}\right)\times 100=\mathrm{Cumulative}\mathrm{Release}\mathrm{of}\mathrm{Risedronate}(\%)$

M_t_ is the amount of cumulative release by time t, and M_∞ _is the total amount of drug present in the carriers. The release profile was finally drawn as cumulative release by time. Release examination was carried out for 30 days, and cumulative release was measured each single day.

**Evaluation of** **swelling and degradation of hydrogel**

The SR of the alginate to alginate/PLGA was measured using the following equation:

$\mathrm{SR}=\frac{\mathrm{Ws}-\mathrm{Wd}}{\mathrm{Wd}}$

Where W_s_ and W_d_ are the weight of the hydrogel in the swollen and dried state, respectively. The SR was measured in time intervals by weighing the disc-shaped hydrogels after immersion in the excess of swelling medium. 

**Cell toxicity assay**

The biocompatibility of ALG, risedronate-loaded ALG (ALG/RIS), and risedronate-containing PLGA microspheres embedded in ALG (ALG/PLGA/RIS) was assessed using the extraction dilution method. Blank PLGA embedded with ALG (ALG/PLGA) was used as the control group. HGFs were suspended in DMEM with 10% fetal bovine serum, 100 µ/ml of penicillin and streptomycin, and 200 mM of L-glutamine and then cultured in a 75-cm^2^ cell culture flask (SPL, South Korea). Cells were incubated at 37 °C and 5% CO_2_, and after 24 hours, unattached cells were rinsed with PBS. The final risedronate concentration in each sample was equal to 0.14 mg/ml. In order to carry out extraction dilution assay, we placed samples in 24-well polystyrene plates. After preparation, the samples were placed in 75% alcohol and were washed three times with PBS. Then 1 ml of cell culture medium was added to each well. Extraction was conducted from each well at 24, 48, and 72 hours. Fibroblast cells (10^4^) in a total volume of 200 µL were counted, diluted and trypsinized and then transferred to 96-well polystyrene plates. Cells were incubated at 5% CO_2_ and saturated humidity at 37 °C for 24 hours. Each assay was repeated five times. After 24 hours, the cell medium was replaced with prepared extractions, and after 24 hours, cell viability was examined by MTT assay. The MTT solution contained tetrazolium yellow crystals, which converted to purple formazan crystals in vital cells. The OD of the resultant solution was determined via spectrophotometer at 570 nm.

MTT assay was carried out by replacing the cell medium with 200 µL of high glucose DMEM; 25 µL of MTT solution was added to each well and incubated for 4 hours. The medium of cells was then completely taken out, and 200 µl of DMSO was added to allow the produced formazan completely dissolved. OD was finally determined by spectrophotometer at 570 nm. The cell viability of fibroblast cells was then calculated using the following formula: 

Cell viability (%)= OD sample/ OD control × 100

**Statistical analysis**

Data were analyzed using one-way ANOVA analysis and Tukey’s test. All statistics were conducted by the aid of SPSS ver. A *p value* less than 0.05 was considered statistically significant. 

## RESULTS

**SEM imaging results**

**Fig. 2:**
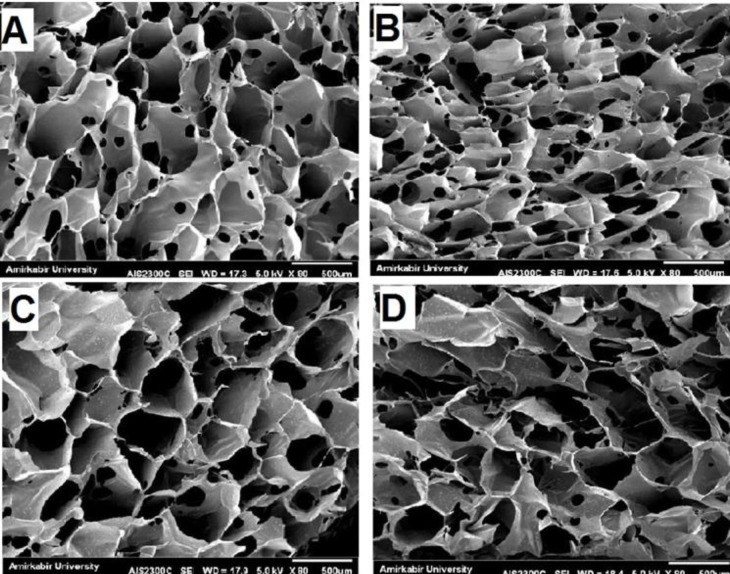
SEM micrograph of (A) ALG ,(B) ALG/RIS, (C) ALG/PLGA and (D) ALG/PLGA/RIS

SEM imaging was performed on ALG, ALG/RIS, ALG/PLGA/RIS, and ALG/PLGA samples to examine surface morphology. Open porosities were observed on hydrogel surface in all samples. These porosities are notable for risedronate release. The hydrogel scaffolds had smooth and homogenous surface in ALG and ALG/RIS samples. Homogenous distribution of microspheres in hydrogel scaffolds was observed after loading PLGA microspheres into ALG (Fig. 2).

**Fig. 3:**
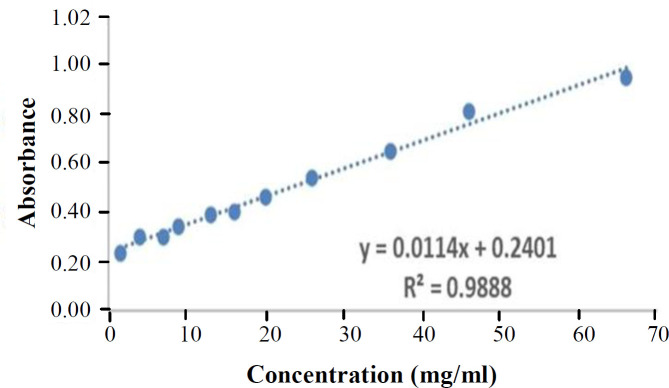
Risedronate calibration curve at 262 nm

**Determination of risedronate absorption and release**

The calibration curve of risedronate was used to determine the amount of risedronate release (Fig. 3). Based on Beer-Lambert law and similar to a previous study the concentrations used were measurable by a UV-Vis spectrophotometer. The absorption of risedronate at the concentrations of 2, 4, 7, 9, 13, 16, 20, 26, 36, 46, and 66 mg/ml was reported as 0.24, 0.30, 0.35, 0.39, 0.41, 0.47, 0.55, 0.65, 0.82, and 0.95, respectively^ [^^17^^]^.

**Encapsulation efficiency**

As the hydrogel was capable of encapsulating risedronate with 100% efficiency, the EE was found to be limited only in PLGA microspheres. To this end, EE was defined and measured only for PLGA microspheres. The mean EE of risedronate in PLGA microspheres after five repeats was equal to 57.14 ± 3.70% (min 53.1% and max 63.01%).

**Cumulative release**

**Fig. 4:**
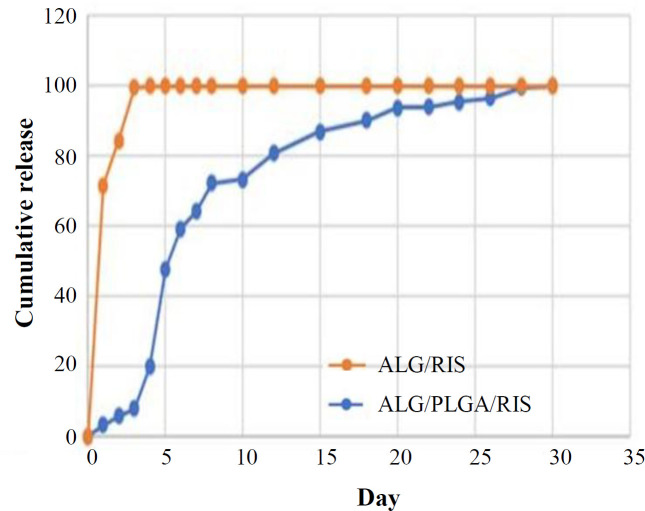
Risedronate cumulative release

The release studies were carried out at 37 °C and pH 7.4. For each group, the release measurement was repeated three times, and the mean amount was reported. Figure 4 shows the cumulative release of risedronate in ALG/RIS and ALG/PLGA/RIS samples. In ALG/RIS group, the burst release of risedronate was observed within first eight hours (67.86% ± 1.90). The remaining risedronate released constantly within 72 hours. By the end of the 3^rd^ day, almost 100% of risedronate was released. In ALG/PLGA/RIS group, after a burst release on 5^th^ day (47.92% ± 2.32), risedronate showed a long and sustained release within the next 23 days. On 28^th^ day, almost 100% of risedronate was released. Within the first three days, the cumulative release of risedronate from ALG/RIS was significantly higher (*p* = 0.000), showing that in the same time, lower amounts of risedronate is released from ALG/PLGA/RIS system.

**Evaluation of** **swelling and degradation of hydrogel**

**Fig. 5:**
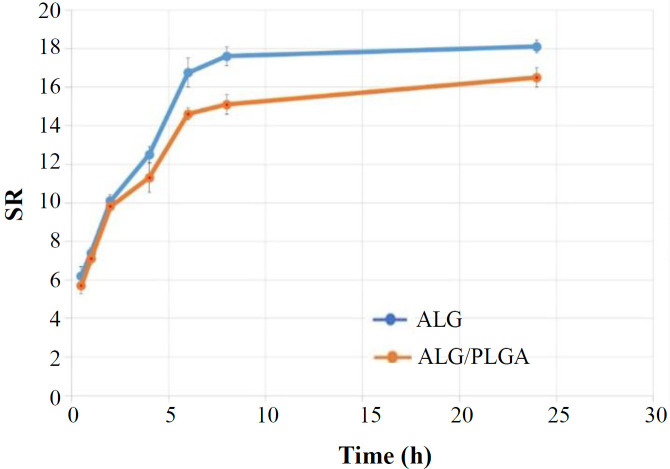
The SR of the alginate and microsphere-loaded hydrogel

The SR of the alginate and microsphere-loaded hydrogel are depicted in Figure 5. After immersion for about 10 hours, the SR of hydrogels remained constant, and the equilibrium was reached. The SR of the alginate was observed to be significantly higher than that of ALG PLGA after a period of six hours and before the end point. The degradation of both ALG and ALG/PLGA was negligible (data not shown).

**Examining cell toxicity**

**Fig. 6:**
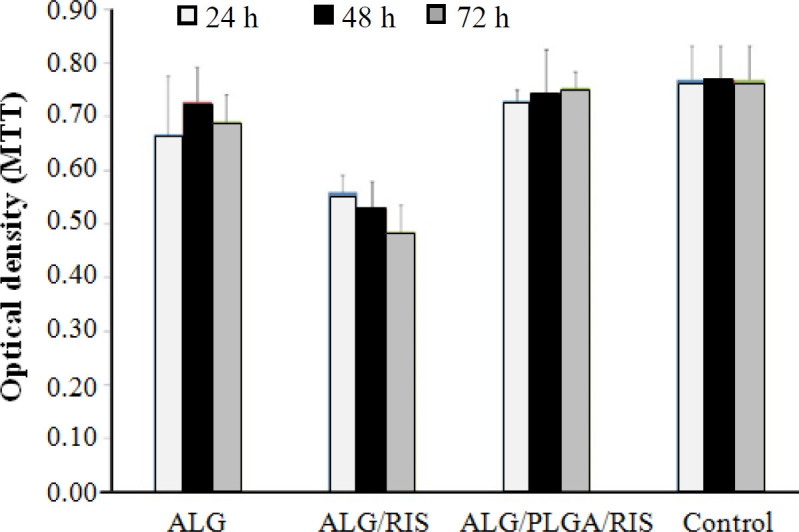
Cell viability of HGF1-P1 cells in ALG, ALG/RIS, ALG/PLGA/RIS and control group

Using the extraction dilution method, the MTT assay was conducted on ALG, ALG/RIS, ALG/PLGA/RIS, and ALG/PLGA, as control group, at 24, 48, and 72 hours. The OD in each group was measured five times with a UV-Vis spectrophotometer at 570 nm, and the mean amount was reported. The cell viability of HGF1-P1 cells was then calculated for each group. Compared to the control group, ALG/PLGA/RIS group did not show significant difference in cell viability (*p* > 0.05); however, both ALG and ALG/RIS showed significant difference in cell viability (*p* < 0.05). According to ISO-10993-5 standard, if the difference between the cell viability on experimental and control groups is higher than 30%, the experimental group is considered toxic^[20]^. In ALG/RIS group, the mean difference in cell viability compared to control group was 72.69% ± 4.08 on 1^st^ day, 68.79% ± 6.31 on 2^nd^ day, and 63.09% ± 6.38 on 3^rd^ day. Considering a difference higher than 30% compared to the control group on 2^nd^ and 3^rd^ days, the ALG/RIS group was regarded to have cell toxicity. Other groups showed differences lower than 30% compared to the control group and were therefore considered to be biocompatible (Fig. 6). 

## DISCUSSION

In this study, we fabricated ALG hydrogel loaded with risedronate and risedronate-containing PLGA microspheres embedded in ALG and also examined their *in vitro* characteristics as a drug delivery system for sustained release of risedronate in local applications. The porous surface of hydrogel and homogenous distribution of PLGA microspheres in hydrogel scaffolds were observed by SEM imaging. After determining EE, the release behavior of risedronate was studied for 30 days. In this regard, ALG/PLGA/RIS system showed slow and sustained release of the drug. The release of risedronate from ALG/RIS system was significantly faster. Cell toxicity examination was carried out using MTT assay with extraction dilution method. The results showed cell toxicity in ALG/RIS system.

SEM micrographs showed open porosities on the surface of ALG. According to the results of Lin *et al.’*s^[^^22^^]^ study, formation of a microporous network in hydrogels is due to the presence of calcium ions in CaCl_2_ aqueous solution. The multivalent calcium ions can form ionic bridges between ALG chains, which leads to the formation of three-dimensional microporous network. The release of the drug can be controlled by decreasing the quantity and size of these porosities. 

The mean of the measured EE of risedronate in PLGA microspheres in the present study was equal to 57.14% ± 3.70. In Kim *et al.*’s^[^^23^^]^ study, PLGA microspheres showed a higher EE because of the porous surface. Compared to our study, the EE in the study of Ryu *et al.*^[^^24^^]^ is notably lower, which is due to the nanosized microparticles in Ryu *et al*’s study^[^^24^^] ^ who previously been shown that decreasing the size of particles leads to higher EE.

Hydrogel-based delivery systems are capable of absorbing high amounts of water, which accelerates the diffusion of small molecules from the gel network^[^^25^^]^; hence, more rapid release of risedronate from ALG/RIS in our study can be explained. Similarly, in Shi *et al.*’s^[^^27^^]^ study, the drug (ibuprofen) was released more rapidly from plain alginate (burst release equal to 85.6%) compared to PLGA/ALG system.

The drug (dexamethasone) release from ALG/PLGA system was in a sustained manner and within 10 hours in the study of Ryu *et al.*^[^^23^^]^. In comparison to the present study, the more rapid release of the drug may be due to the nanometer size of particles. In this regard, the decreased particle sizes may lead to an increase in surface area between particles and, subsequently, increases the release rate of drug out of the microspheres^[^^27^^]^. Lin *et al.*^[^^21^^]^ utilized PLGA microspheres embedded in homogenous ALG (alginate hydrogel) for releasing magnesium ions, which effectively decreased the amount of burst release and resulted in a long and controlled release. ALG was used in two different concentrations (3% and 5%). The higher concentration of ALG led to more controlled drug release. Based on the results of this study, increasing alginate concentration decreases the quantity and size of surface porosities and, subsequently, decreases the amount of the drug released. The more effectiveness of ALG/PLGA system, compared to our study, may be because of higher concentration of alginate (3% and 5% versus 2%).

The concentration of CaCl_2_ used for alginate cross-linking in the present study was equal to 100 mM. Mandal *et al.*^[^^28^^]^ showed that the higher concentration of CaCl_2_ cross-linking concentration leads to the formation of a harder gel network and, consequently, more sustained release behavior in alginate microspheres. According to Zhai et al.’s^[^^29^^]^ study, high concentrations of CaCl_2_ (100 mM and 50 mM) adversely affected the cell viability of RSC96, 3T3, and L8 cell lines, which was mainly due to free calcium ions. It is speculated that CaCl_2_ concentration is required to be optimized in order to provide enough gelation, while avoiding the cell toxicity of free calcium ions. 

In our study, the concentration of risedronate used in cell toxicity examinations was equal to 0.14 mg/ml. This concentration caused cell toxicity only in ALG/RIS group, which may be due to the burst release of risedronate within the first three days. Nasr *et al.*^[^^30^^]^ examined risedronate cell toxicity on Calu-3 cells. According to their results, risedronate did not decrease cell viability in concentrations lower than 0.5 mg/ml and, therefore, was not toxic in concentrations similar to our study^[^^31^^]^.

In summary, PLGA microspheres embedded in ALG are capable of controlled, slow and sustained release of risedronate and do not show cell toxicity on gingival fibroblasts. This drug delivery system can serve as an appropriate system for risedronate drug delivery and in local applications.

## DECLARATIONS

### Ethical statement

The above-mentioned sampling protocols were approved by Research Ethics Committee of Islamic Azad University, Dental branch, Tehran, Iran (ethical code: IR.IAU.DENTAL.REC.1399.151).

### Data availability

The analyzed data sets generated during the study are available from the corresponding author on reasonable request.

### Author contributions

SA, conceptualization, supervision, project administer

ation, data curation; ES, writing the manuscript; GA, writing original draft and funding acquisition.

### Conflict of interest

None declared.

### Funding/support

No funding or support was received for this study.
